# Prognostic Nomogram Predicting Survival and Propensity Score Matching with Demographics and Comparative Analysis of Prostate Small Cell and Large Cell Neuroendocrine Carcinoma

**DOI:** 10.3390/jcm13164874

**Published:** 2024-08-18

**Authors:** Asad Ullah, Abdul Qahar Khan Yasinzai, Kue Tylor Lee, Tristin Chaudhury, Hannah Chaudhury, Abdullah Chandasir, Agha Wali, Abdul Waheed, Bisma Tareen, Marjan Khan, Aman Goyal, Asif Iqbal, Amir Humza Sohail, Soban Maan, Abu Baker Sheikh, Sayed Ab Reshad Ghafouri, Israr Khan, Jaydira Del Rivero, Nabin R. Karki

**Affiliations:** 1 Texas Tech University Health Sciences Center, Lubbock, TX 79430, USA; tristin.chaudhury@ttuhsc.edu (T.C.); hannah.chaudhury@ttuhsc.edu (H.C.); 2 University of Florida Health Cancer Center, Gainesville, FL 32608, USA; a.yasinzai@ufl.edu; 3 Medical College of Georgia, Augusta, GA 30912, USA; kuleee@augusta.edu (K.T.L.); achandasir@augusta.edu (A.C.); 4 Bolan Medical College, Quetta 83700, Pakistan; akwali092@gmail.com (A.W.); bismatareen@hotmail.com (B.T.); 5 Department of Surgery, Baycare Health System, Clearwater, FL 33759, USA; abdul.waheed@baycare.org; 6 Marshfield Clinics, Marshfield, WI 54449, USA; khan.marjan@marshfieldclinic.org; 7 Seth GS Medical College and KEM Hospital, Mumbai 400012, India; amanmgy@gmail.com; 8 Mercy Hospital, Ardmore, OK 73401, USA; asif702@gmail.com; 9 Department of Surgery, University of New Mexico, Albuquerque, NM 87106, USA; ameer.hamzasohail@gmail.com; 10 Department of Internal Medicine, West Virginia University, Morgantown, WV 26506, USA; 11 Department of Internal Medicine, University of New Mexico, Albuquerque, NM 87131, USA; abubaker.sheikh@gmail.com; 12 Department of Hematology-Oncology, Texas Tech University Health Sciences Center, Lubbock, TX 79430, USA; s.reshad.ghafouri@ttuhsc.edu; 13 Department of Medicine, Insight Hospital and Medical Center, Chicago, IL 60616, USA; israr.khan@insightchicago.com; 14 Division of Medical Oncology, National Institute of Health (NIH), Bethesda, MD 20814, USA; jaydira.delrivero@nih.gov; 15 Mitchell Cancer Institute, University of South Alabama, Mobile, AL 36604, USA

**Keywords:** prostate cancer, small-cell neuroendocrine carcinoma, large-cell neuroendocrine carcinoma, SEER, chemotherapy, prognostic nomogram

## Abstract

**Background**: This retrospective study aims to examine the patient demographics, survival rates, and treatment methods for small-cell neuroendocrine carcinoma (SCNEC) and large-cell neuroendocrine carcinoma (LCNEC) of prostate origin while also identifying the main differences between common types of prostate cancer with comparative analysis for survival. **Methods**: Our analysis utilized the Surveillance, Epidemiology, and End Results database (SEER), and data was collected from 2000–2020. Cox proportional hazards and chi-squared analysis were used for statistical analysis. **Results**: A total of 718 cases of prostate small and large neuroendocrine carcinoma were identified. The median age was 71.5 years, and the median follow-up was 11.0 years (95% confidence interval (95% CI) = 9.2–12.8). Most patients were over the age of 80 years (33.8%) and Caucasian (74.4%). The overall 5-year survival was 8.0% (95% CI = 6.8–9.2). The 5-year OS for Caucasians was 7.3% (95% C.I. 6.0–8.3). For Black Americans, the 5-year OS was 11.9% (95% C.I. 7.3–16.5). For Hispanics, the 5-year OS was 12.2% (95% C.I. 7.7–16.7). The 5-year cause-specific survival (CSS) was 16.2% (95% CI = 14.3–18.1). For treatment modality, the five-year survival for each were as follows: chemotherapy, 3.5% (95% CI = 2.1–4.9); surgery, 18.2% (95% CI = 13.6–22.8); multimodality therapy (surgery and chemotherapy), 4.8% (95% CI = 1.7–7.9); and combination (chemoradiation with surgery), 5.0% (95% CI = 1.0–9.0). The prognostic nomogram created to predict patient survivability matched the findings from the statistical analysis with a statistical difference found in race, income, housing, stage, and nodal status. The nomogram also indicated a slight increase in mortality with tumors of greater size. This analysis showed a slight increase in mortality for patients of Asian race. In addition, there was a significant increase in death for patients with stage 3 tumors, as well as patients who underwent surgery and radiation. Furthermore, we performed propensity score matching for survival differences, and no survival difference was found between SCNEC and LCNEC. **Conclusions**: Asian patients, larger tumor size, and distant disease were associated with worse long-term clinical outcomes. By leveraging insights from registry-based studies, clinicians can better strategize treatment options, improving patient outcomes in this challenging oncology arena.

## 1. Introduction

Prostate cancer stands as one of the most frequently diagnosed malignancies in men worldwide. Prostate cancer is the fifth leading cause of death worldwide and the second most frequent cancer diagnosis made in men [1]. It is a disease with incidence rates varying significantly worldwide ranging from 6.3 to 83.4 per 100,000 people [1,2]. High-grade neuroendocrine carcinoma typically emerges in the context of previous anti-androgen treatment, despite it having the potential to arise de novo [3]. Additionally, more than 95% of cases of prostate cancer emerge as adenocarcinoma [4]. Neuroendocrine prostate cancer (NEPC) is categorized into many histological subtypes; however, small-cell neuroendocrine carcinoma (SCNEC) and large-cell neuroendocrine carcinoma (LCNEC) are focused on in this study [5,6]. These subtypes pose significant clinical challenges due to their aggressive behavior, resistance to conventional treatments, and poor prognosis [6]. Understanding their prognosis is crucial to inform therapeutic decisions and improve patient outcomes. 

The SEER studies spanning multiple years have contributed to our understanding of the epidemiology and prognosis of these rare subtypes of prostate cancer [7,8,9]. These studies underscore the rarity of the neuroendocrine subtypes but also emphasize their aggressive nature and the consequent poor survival outcomes, necessitating the development of effective prognostic tools and therapeutic strategies [8,9]. 

Some several key biomolecules and pathways are implicated in metastatic prostate cancer progression and therapeutic resistance. Prostate-specific antigen (PSA) is a widely used biomarker that, although not without limitations, aids in screening and monitoring disease status [10]. PSA levels correlate with tumor burden and the extent of disease in typical prostate cancer [10,11]. The androgen receptor (AR) is a key transcription factor that drives prostate cancer growth in response to androgenic hormones and is a regulator in castration-resistant prostate cancer (CRPC) [12]. Additionally, the homeobox protein NKX3-1, which serves as a prostate lineage-specific tumor suppressor involved in regulating prostate tissue differentiation, is recommended to ascertain prostatic origin in doubtful cases due to its high, though not exclusive, specificity for prostate tissues [13]. 

In contrast to prostate adenocarcinoma, a more common cancer, both SCNEC and LCNEC of the prostate lack significant PSA production and AR signaling [14]. Although LCNEC may express PSA or AR to some extent, the levels are typically absent or lower than those found in high-grade prostate adenocarcinoma, helping differential diagnosis [15]. It also has been found that SCNEC and LCNEC fail to express NKX3-1 [16,17]. The loss of AR and NKX3-1, along with neuroendocrine transdifferentiation, are key contributors to the highly aggressive clinical behavior of prostatic SCNEC/LCNEC [16,17]. This includes resistance to standard androgen deprivation therapy, as well as higher rates of metastasis and poorer survival compared to localized prostate adenocarcinoma [18,19]. Together, aberrations in PSA levels, AR signaling, and NKX3-1 expression provide growth advantages to prostate cancer cells, while also serving as useful biomarkers of disease status and potential therapeutic targets [19].

Recognizing the clinical challenges posed by SCNEC and LCNEC, this study aims to delve deeper into these challenging subtypes of neuroendocrine prostate cancer by leveraging insights from epidemiological data and clinical outcomes analysis. Understanding these molecular distinctions not only aids in differential diagnosis but also opens avenues for the development of targeted therapies. By establishing accurate prognostic tools and exploring new therapeutic strategies, this research aims to significantly enhance clinical outcomes and improve the quality of life for patients afflicted with these rare and aggressive prostate cancer subtypes. This study aims to develop a comprehensive understanding of neuroendocrine prostate cancer, particularly focusing on its two rare histological subtypes: SCNEC and LCNEC. Given the aggressive nature of these rare cancers, it is important to establish accurate prognostic tools and effective therapeutic strategies. This study aims to contribute to the enhancement of clinical outcomes and the tailoring of treatment approaches of SCNEC and LCNEC and support the pursuit of targeted therapies that could improve survival rates and quality of life.

## 2. Methods

The Surveillance, Epidemiology, and End Results (SEER) was initiated by the National Cancer Institute in 1973. This population-level database covers 26.5% of cancer patients in the United States. Data from 2000 to 2020 was collected using SEER*Stat software (Version 8.4.2) which encompasses 17 SEER registries. The International Classification of Diseases Version 3 (ICD-I-3) was used. This data was then exported to Statistical Package for Social Sciences (SPSS, IBM, Armonk, NY, USA) Version 28.0.0.0 for analyses.

**Figure 1:**
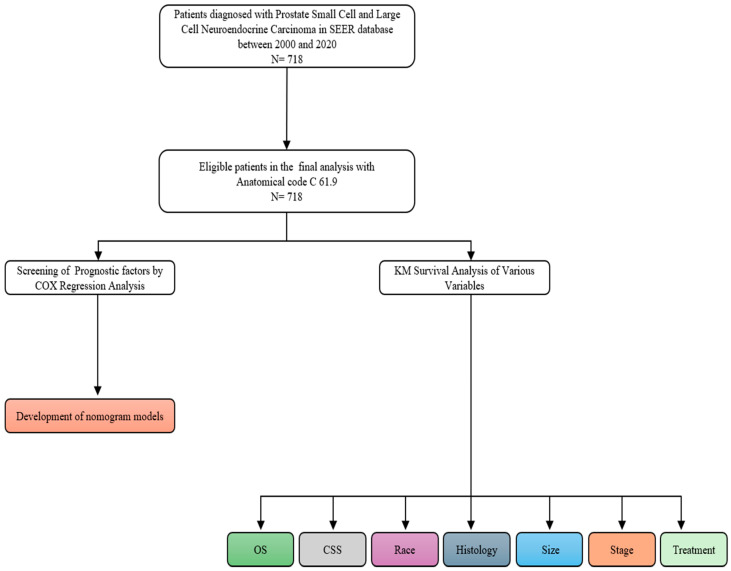
Exclusion flowchart.

This study used demographic factors, tumor characteristics, and other variables including age, race, median income, housing, histology, tumor size, tumor stage, lymph node status, metastasis, and treatment modalities (surgery, radiation, chemotherapy)—factors that have been to affect the survival of various cancers. The stage is defined differently by SEER compared to the Union for International Cancer Control TNM (“Tumor”, “Nodes”, “Metastases”) staging. “Localized” is defined as tumors confined to the organ of origin. The “Regional” stage encompasses cases in which cancer spreads via direct extension to adjacent organs or tissues and/or spread to lymph nodes considered regional to the organ of origin, but no further spread has occurred. “Distant” is denoted as cases where cancer has spread beyond adjacent organs or tissues, and/or metastasis to distant lymph nodes or tissues. These guidelines and criteria were chosen based on previously published and validated studies (https://scdhec.gov/CancerRegistry/Definitions https://training.seer.cancer.gov/staging/systems/ accessed 14 November 2023) (Figure 1).

Demographic statistics, survival factors, and survival trends were analyzed using SPSS. Cox proportional hazards regression analysis was utilized to examine associations among demographic factors, tumor characteristics, treatment modality, and overall survival. Categorical data were expressed as proportions and analyzed using the chi-squared test. Hazard ratios (H.R.) calculated by the Cox model for continuous variables were calculated by the study endpoint with a predefined unit of increase of the independent variable. Log-rank *p* values, H.R., and 95% confidence intervals (C.I.) were calculated. Associations and comparisons were visualized on a graph via generating Kaplan-Meier survival plots. The underlying assumptions of the multiple linear regression models, including linearity and normality of residuals, were thoroughly evaluated to ensure the validity and reliability of the statistical inferences drawn from the analysis. Endpoints included overall survival and disease-specific survival. Multivariate analysis was used to identify independent risk factors. Univariate and multivariate survival analysis was performed to view such associations and assess for independence from known demographic, clinical, and treatment variables. Cases with unknown variables in multivariate analysis were censored. A two-tailed *p*-value < 0.05 was considered significant. A prognostic nomogram was also constructed to be used to predict future patient mortality and survival rate. This was done through the “rms” package in R.

### Nomogram Creation and Propensity Score Matching

After pertinent prognostic variables were identified, the resultant mathematical models were then used to construct a nomogram. By giving each variable a numerical score determined by how much of an impact it has on the overall forecast, the nomogram provides a visual representation of the predictive model. Following the addition of these scores, a total point value is produced that is correlated with the expected likelihood or risk of the specified outcome, such as the course of the disease or survival. The correctness and generalizability of the nomogram were confirmed through the use of internal and external validation approaches. Internal validation entailed evaluating the nomogram’s performance within the original dataset using resampling techniques like bootstrapping or cross-validation. To evaluate the performance and reliability of a nomogram, it was subjected to r testing and validation using receiver operating characteristic (ROC) curves and area under the curve (AUC) metrics. Initially, the nomogram was applied to a training dataset to derive the predictive model and generate initial ROC curves. These curves were then analyzed to assess the model’s ability to discriminate between different outcomes, with the AUC providing a quantitative measure of overall performance. To ensure robustness, the nomogram was subsequently validated using an independent test dataset. This validation involved recalculating the ROC curves and AUC for the test dataset to determine how well the nomogram generalized to new, unseen data.

To assess the difference in survival between small cell and large cell carcinoma patients, propensity score matching was performed using a nearest-neighbor matching algorithm. The matching process was conducted in a stepwise manner. First, individuals in the small cell group were matched to the nearest neighbor in the large cell group based on their propensity scores. The propensity score was estimated using logistic regression based on age, race, primary site, income, and housing standardized mean differences and the distribution of propensity scores were analyzed to ensure that the matching process successfully reduced the imbalance between the small and large cell groups. After performing propensity score matching, the survival was compared between the two groups. Paired t-tests were used to analyze the matched data and estimate the treatment effect.

## 3. Results

In this study, 718 cases of prostate small and large neuroendocrine carcinoma were identified.

### 3.1. Demographic Data

Among the patients in this study, the median age was 71.5 years. The median follow-up was 11.0 years (9.2–12.8).

The most common age band was 70–79 years (33.8%) followed by 60–69 years (28.3%). All patients in this cohort were male. Regarding race, the majority of cases were White (74.4%), followed by Hispanic (9.5%), then Black (9.2%). The median household income in the United States in 2022 was $74,580 (https://www.census.gov/library/publications/2023/demo/p60-279.html accessed on 20 March 2024). We divided the household income into upper and lower quartiles. The median income of the majority of this cohort was ≤$75,000 (54.0%), while the minority was ≥$75,000 (46.0%). Rural-Urban Continuum Codes distinguish metropolitan (metro) counties by the population size of their metro area, and nonmetropolitan (nonmetro) counties by degree of urbanization and adjacency to a metro area or areas (https://seer.cancer.gov/seerstat/variables/countyattribs/ruralurban.html, accessed on 20 May 2024) The majority of this cohort was in metropolitan areas (618, 86.1%) and the minority lived in rural areas (13.9%) (Table 1).

### 3.2. Tumor Characteristics

The histology of the majority of cases were small-cell neuroendocrine carcinoma (95.8%), while the minority was large-cell neuroendocrine carcinoma (4.2%). Tumor size was known in 89 (12.4%) cases, of which 15 (16.9%) were ≤2 cm, 13 (14.6%) were 2.1 cm to 4.0 cm, and 61 (68.5%) were >4.0 cm. Lymph node status was known in 58 (8.1%) cases, of which 30 (51.7%) had positive lymph nodes. Metastases status was fully known (to bone, brain, liver, lung, distant lymph node, and other) in 292 (40.7%) cases, of which 211 (27.7%) had a positive metastases status. Of these, metastases were to a single site (57.9%) (Table 2).

**Figure 2:**
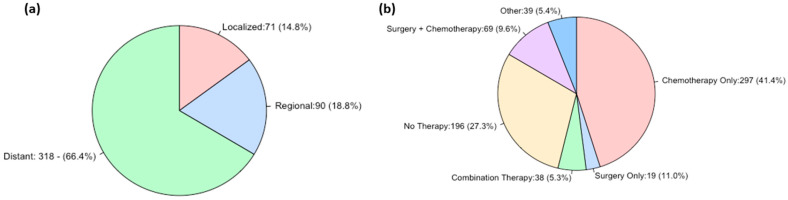
Pie chart of tumor stage (**a**) and treatment modality (**b**) (*n* = 479, 66.7% of cohort); Other = radiation only, radiation and chemotherapy, surgery and radiation; +Combination: chemotherapy, radiation therapy, and surgery treatment.

Extent of the disease was unknown in 239 (33.3%) cases. In the remaining (66.7%), cases where the extent of disease was known, most cases (66.4%) were distant, followed by regional (18.8%), then localized (14.8%) (Figure 2a).

### 3.3. Treatment Modality

Most cases in this cohort underwent chemotherapy only (41.4%), followed by surgery only (11.1%), then surgery and chemotherapy (9.6%). No cases received radiation only. Thirty-eight (5.3%) cases underwent combination therapy (surgery, radiation, and chemotherapy). One hundred and ninety-six (27.3%) cases did not undergo therapy (Figure 2b). Of all cases, 302 (42.1%) had an unknown chemotherapy status (Table 3).

### 3.4. Outcomes and Survival Analysis of Overall and Cause-Specific Survival

**Figure 3:**
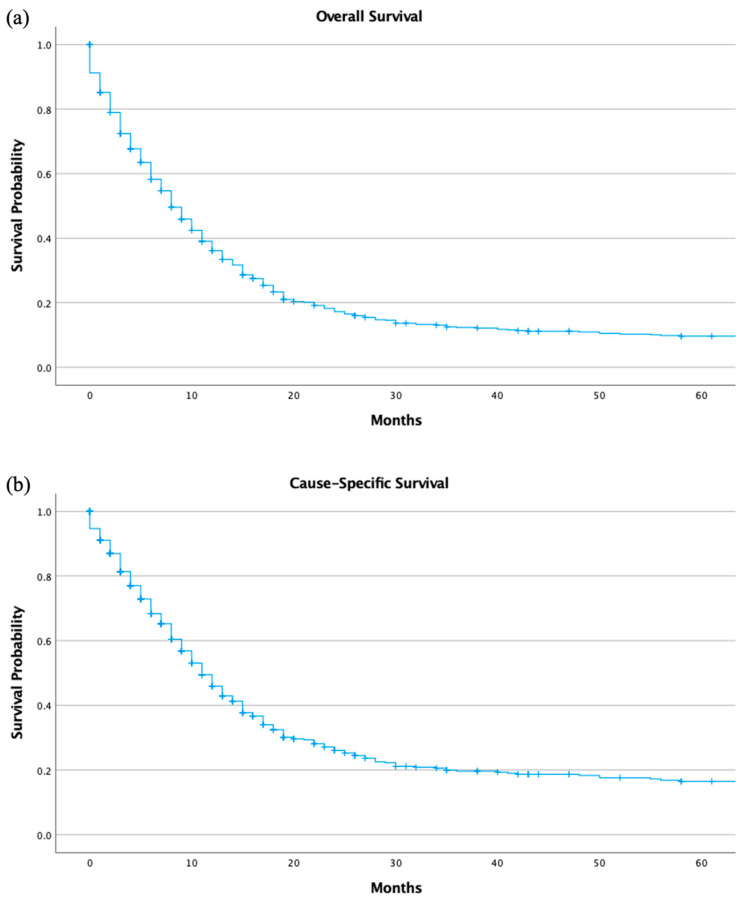
(**a**) Overall survival (OS) and (**b**) Cause-specific survival (CSS).

The 5-year overall survival of the study group was 8.0% (95% C.I. 8.0% (6.8–9.2)). SEER calls disease-specific survival as “cause-specific survival” in their database. The 5-year cause-specific survival (CSS) was 16.2% (95% C.I. 14.3–18.1) (Supplementary Materials Table S1). The Kaplan-Meier graph visualizing the 5-year survival is listed below (Figure 3a,b). The 1-year and 5-year survival with chemotherapy only was 15.5% (95% C.I. 13.2–17.8) and 3.5% (95% C.I. 2.1–4.9), respectively. The 1-year and 5-year survival with surgery only with an unknown chemotherapy status was 24.0% (95% C.I. 19.9–29.9) and 18.2% (95% C.I. 13.6–22.8), respectively. The 1-year and 5-year survival with surgery and chemotherapy was 12.0% (95% C.I. 7.7–16.3) and 4.8% (95% C.I. 1.7–7.9), respectively. The 1-year and 5-year survival with combination therapy was 31.7% (95% C.I. 23.7–39.7) and 5.0% (95% C.I. 1.0–9.0), respectively (Supplementary Materials Table S2). The survival analysis of other therapies not included in this analysis could not be performed due to a limited number of cases.

### 3.5. Outcomes and Overall Survival of Different Treatment Modalities

**Figure 4:**
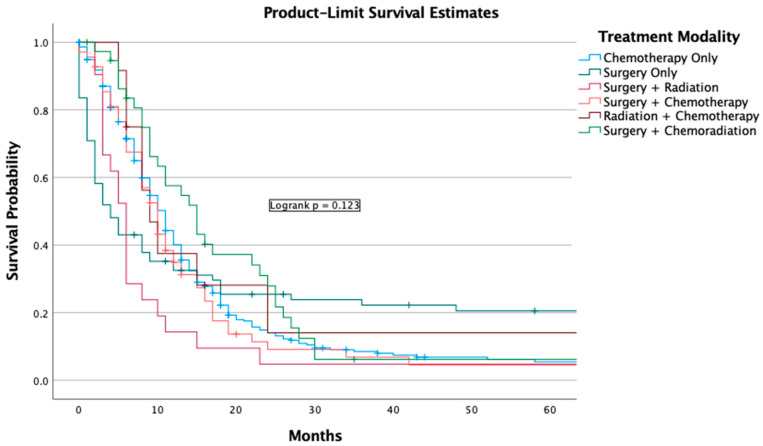
Survival analysis of treatment modality.

There was a statistically significant difference (*p* = 0.042) among combination therapy, no therapy, chemotherapy only, surgery only, surgery + chemotherapy, and surgery + radiation (Figure 4).

### 3.6. Outcomes and Overall Survival of Demographic Factors

Univariate analysis indicated that increased age was a negative predictor (1.02 (1.01–1.03), *p* < 0.001) (Table 3).

The 1-year overall survival for Caucasians was 17.2% (95% C.I. 15.5–18.9) and 5-year OS was 7.3% (95% C.I. 6.0–8.3). For Black Americans, the 1-year survival was 22.6% (95% C.I. 17.0–28.2) and 5-year OS was 11.9% (95% C.I. 7.3–16.5). For Hispanics, the 1-year survival was 28.1% (95% C.I., 22.2–34.0) and 5-year OS was 12.2% (95% C.I. 7.7–16.7). Asian or Pacific Islander and American Indian or Alaska natives’ overall survival could not be calculated due to limited power. Race was considered a predictor for survival (*p* = 0.026) (Figure 4. Univariate analysis revealed that Hispanics were associated with positive prognosis (*p* = 0.005) (Table 3). There was no significant difference in survival with different income levels (Supplementary Materials Figure S1a). Housing status was not associated with worse survival outcomes (Supplementary Materials Figure S1b).

### 3.7. Outcomes and Overall Survival of Tumor Characteristics

**Figure 5:**
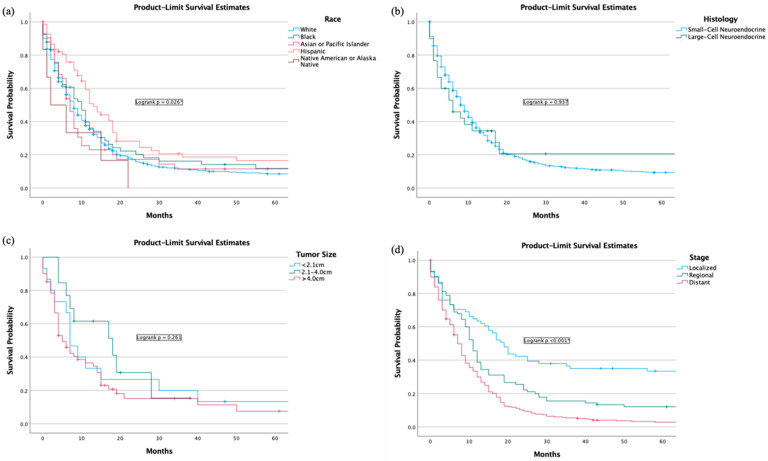
Overall survival analysis by (**a**) race, (**b**) histology, (**c**) tumor size, and (**d**) tumor stage. * Represents significance, *p*-value < 0.05.

SCNEC or LCNEC histologic subtype was not found to be a prognostic predictor (Figure 5b).

The higher tumor stage (Figure 5d) was found to be an adverse prognostic predictor (*p* < 0.001). Univariate analysis revealed regional stage (1.70 (1.21–2.39), *p* = 0.002) and distant dissemination of disease (2.63 (2.00–3.53), *p* < 0.001) were considered negative predictors (Table 2).

Larger tumor size (Figure 5c) was not found to be prognostic.

Nodal status (Supplementary Materials Figure S1c) was found to be a prognostic factor (*p* < 0.001). Univariate analysis revealed that a positive nodal status (2.81 (1.49–5.30), *p* < 0.001) was associated with worse outcomes (Table 2).

Metastases to more than one site compared to an isolated site were not a prognostic factor (Supplementary Materials Figure S1d).

### 3.8. Propensity Score Matching for Large and Small Cell Carcinoma

Propensity score matching was used to determine the difference between SCNEC and LCNEC on survival. Each LCNEC patient (N = 60) was matched to a small cell carcinoma patient. Acceptable standardized mean differences were achieved with most being around 0.1–0.3. As found by the univariate analysis, there was no statistically significant difference in survival outcomes between histological types (*p* = 0.6353)

### 3.9. Prognostic Nomogram Construction

**Figure 6:**
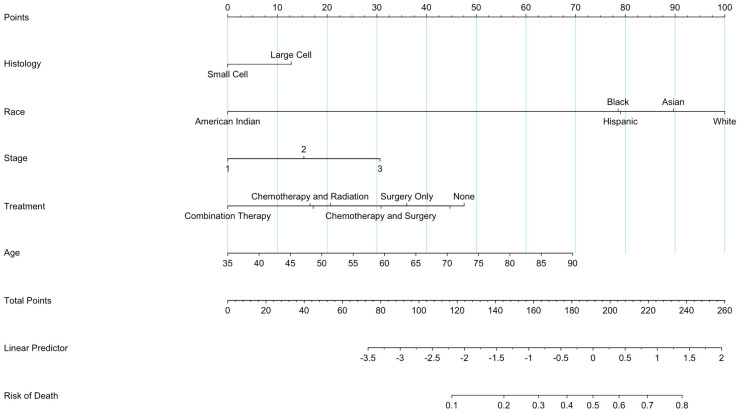
Prognostic nomogram for SNCEC and LCNEC of the prostate.

To construct a model upon which patient survivability could be predicted, a prognostic nomogram was created (Figure 6). The prognostic nomogram indicated that there was minimal difference in survival between patients with SCNEC and LCNEC. There was a slight increase in mortality for patients of Asian race. In addition to that, there was a significant increase in death for patients with stage 3 tumors, as well as patients who underwent surgery and radiation. The nomogram also indicated a slight increase in mortality with tumors of greater size. The propensity score analysis demonstrated good covariate balance across the groups, indicating the groups were well-matched on observed confounders. Specifically, standardized mean differences for all covariates were less than 0.1, suggesting adequate balance. Sensitivity analyses confirmed the robustness of these associations to alternative modeling assumptions, indicating good sensitivity of the analysis.

The nomogram is utilized by assigning each categorization/variable a point value based on its alignment to the top “points” bar. For a given patient, the point values assigned based on histology, race, stage, treatment, size, and age are then added up. That value is then tracked on the “Total Points” bar, and the risk of death in 5 years is calculated.

**Figure 7:**
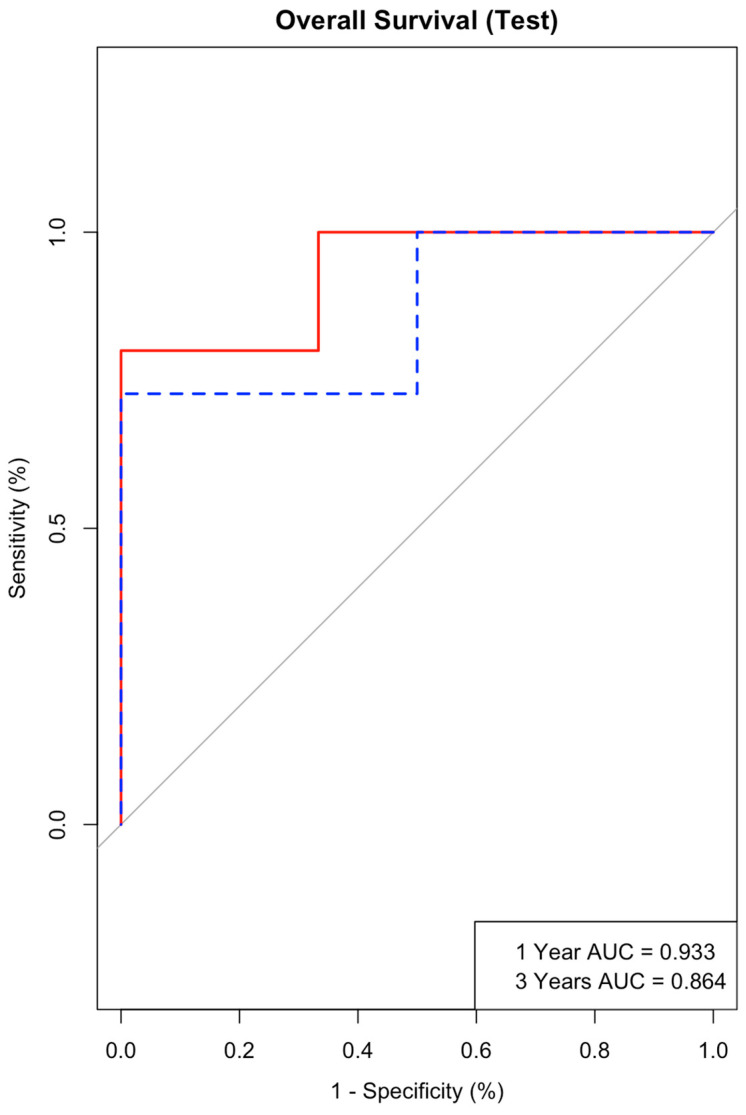
Test AUC curve for SCNEC and LCNEC of the prostate.

The AUC values for the training cohort were 0.954 for 1 year and 0.911 for 3 years. The AUC values for the test cohort for 1 year and 3 years were 0.933 and 0.864, respectively (Figure 7). A high AUC value in both the training and test phases indicated that the nomogram had strong discriminatory power and was effective in predicting the outcomes of interest. Additionally, calibration plots were examined to assess how well predicted probabilities matched observed outcomes, further confirming the nomogram’s accuracy and reliability.

## 4. Discussion

The study provides in-depth analysis of prostatic SCNEC and LCNEC. This retrospective population-based study highlights vital prognostic factors, thus making a significant contribution to the process of clinical decision-making. Prognostic factors are used commonly as predictors of survival in various studies. For example, in an investigation analyzing prostate cancer, men of African ancestry had worse outcomes compared to men of European ancestry [20]. Within our study, the prognostic nomogram indicated that there was minimal difference in survival between patients with SCNEC and LCNEC. There was a slight increase in mortality for patients of Asian race. In addition, there was a significant increase in death for patients of stage 3 tumors, as well as patients who underwent surgery and radiation. The nomogram also indicated a slight increase in mortality with tumors of greater size.

We can first look at the demographics related to prostate cancer in other studies and compare them to those found in our study. With regard to age, in general, it is uncommon to find prostate cancer in men under the age of 55 years [21]. Siegel et al. (2020) found that the incidence of prostate cancer was greatest for men in the 70–74 years old range [22]. Our demographic data examining SCNEC and LCNEC of the prostate revealed a median age of 71.5 years for patients in this study. Therefore, the median age in our study adheres to the ages found in the previously mentioned studies.

Another demographic factor tied to prostate cancer that can be examined is race. One study claimed that the incidence and mortality rates of prostate cancer vary according to race. As a result, they sought to determine if there were differences in the expression of neuroendocrine cells in normal prostates among different ethnic groups. The study found neuroendocrine cell expression was significantly higher in Asians and Whites compared to African Americans [23]. These demographic findings of a neuroendocrine cell expression study resemble the demographics of our SCNEC and LCNEC study in that the white population is part of the majority affected and African Americans were the least affected. Race breakdown identified a White majority followed by Hispanics then African American. 

Lastly, we can compare the demographics—specifically race and age—of common prostate cancers such as adenocarcinoma to our study on SCNEC and LCNEC. A study analyzing prostate adenocarcinoma identified white patients accounting for the majority of cases (71.2%), which aligns with our findings. In addition, this study found the median age was 52 years old which contrasts with our small cell and large cell neuroendocrine prostate cancer median age of 71.5 years of age [24].

Another important element to explore are the reports of the survival rates of prostate cancer. Several studies have viewed the 5-year and 1-year overall survival rate for prostate small cell carcinoma. In a case series analyzing Chinese patients with small cell carcinoma of the prostate, the 1-year survival rate was 23.2% [25]. This contrasts with a population-based investigation in the US using the SEER database that evaluated the current trend of prostate small cell carcinoma worsening prognosis. The survival rates observed were 12.5% for 5-year and 42.1% for 1-year both of which are greater than our comprehensive study [7]. In fact, in our own investigation, the 5-year overall survival rate for both prostate small cell and large cell neuroendocrine carcinomas stood at 8.0% (95% CI = 6.8–9.2) while the 1-year overall survival rate was 18.5% (95% CI = 17.0–20.0).

The survival rates of SCNEC and LCNEC can be compared to a more common prostate cancer, adenocarcinoma, by which the survival metrics differ. For example, Siegel et al. (2020) described a 5-year survival for distant-stage prostate adenocarcinoma that improved from 28.7% during 2001–2005 to 32.3% during 2011–2016 [22]. Thus, there is a huge contrast in the 5-year survival rate between the rare SCNEC and LCNEC (8.0%) and the more common prostate adenocarcinoma (28.7–32.3%). The aforementioned study highlighted a 5-year survival of the following races: Asian/Pacific Islanders (API) (42.0%), followed by Hispanics (37.2%), American Indian/Alaska Natives (AI/AN) (32.2%), Black men (31.6%), and White men (29.1%) [22]. Our findings suggest similar trends on race for a 5-year survival rate: Hispanic (12.2%), Black (11.9%), then White (7.3%).

The last major factor to consider when discussing prostate cancer is treatment modality. Various studies have noted certain survival rates with specific treatment modalities. In a recent study, neuroendocrine prostate cancer survival outcomes were compared with different therapeutic modalities (chemotherapy, radiation therapy, and surgery), and Zhu et al. (2021) noted chemotherapy was the most effective therapy. Chemotherapy increased the patient survival outcome with regional and distant metastases from 8 months and 5 months, respectively, to 13.5 months and 9 months, respectively [9]. This study contrasts with our findings of chemotherapy being the least effective treatment modality for a 5-year survival rate (3.5%) and the second to least effective treatment modality for a 1-year survival rate (15.5%). In further examination of the literature, Tzou et al. (2018) found that chemotherapy with etoposide and cisplatin is effective to achieve remission in LCNEC and surgery post-chemotherapy may be considered [26]. In contrast to these findings, our results found that combined surgery and chemotherapy has a 4.8% 5-year survival rate, one of the less effective therapeutic modalities in our findings. Moreover, our cohort underwent several treatment modalities with the following 5-year survival rates: surgery only (18.2%), combination (chemoradiation with surgery) (5.0%), surgery and chemotherapy (4.8%), and chemotherapy only (3.5%).

Once more, we can compare the therapeutic modalities for the common prostate cancer, adenocarcinoma, to our study findings. One study looked at the 5- and 10-year survival rates of 120 men over 13 years. The findings suggest radical prostatectomy to be the best therapy with a 5- and 10-year survival rate of 97% and 62%, respectively [27]. In our findings, treatment with surgery only had survival rates of 24.0% and 18.2% for 1-year and 5-year survival rates, respectively. In addition, Underwood et al. (2005) identified 142,340 men of different racial backgrounds and found an increase in utilization of radical prostatectomy and combination therapy [28]. The increased usage of these therapies follows the trends of our findings with combination therapy having the highest 1-year survival rate at 31.7% and surgery only having the highest 5-year survival rate at 18.2%.

The findings of this study underscore several critical clinical implications for the management of SCNEC and LCNEC. Identifying prognostic factors such as tumor stage, patient age, and treatment modality can significantly enhance clinical decision-making, guiding clinicians in tailoring more aggressive or alternative therapeutic strategies for high-risk patients. Demographic insights, including the higher incidence of these subtypes in older patients and certain racial groups, should inform screening and monitoring programs, potentially improving survival outcomes through early detection and personalized treatment plans. The stark contrast in survival rates between neuroendocrine subtypes and more common prostate cancers, such as adenocarcinoma, highlights the urgent need for novel therapeutic approaches, as conventional treatment protocols may be inadequate. Future research should focus on the detailed molecular characterization of SCNEC and LCNEC to identify therapeutic targets and explore the potential of immunotherapy, particularly in combination with existing treatments. Longitudinal and multi-center studies would enhance the robustness of findings and facilitate the generalization of results across diverse populations. Personalized medicine approaches utilizing genomic and proteomic profiling could lead to more effective and less toxic treatment regimens, ultimately improving quality of life and survival rates. Addressing these research directions can lead to significant strides in the fight against SCNEC and LCNEC, resulting in better patient outcomes and more effective management strategies for these challenging cancer subtypes.

## 5. Limitations

The study has certain limitations that should be acknowledged. The retrospective design and dependence on the SEER database, while comprehensive, do not provide detailed information regarding patients’ comorbid conditions, functional status, the exact chemotherapy used, or the order in which treatments were administered. Additionally, the grouping of SCNEC and LCNEC for this analysis does not account for the potential differences in their genetic profiles and clinical courses. Our study likely includes secondary NEPC (treatment-related neuroendocrine prostate cancers or aggressive-variant prostate cancers) along with the primary NEPC (pure neuroendocrine carcinomas of the prostate present at diagnosis) each of which has different tumor biology and clinical outcomes [29,30].

## 6. Conclusions

Our study provides a comprehensive analysis of SCNEC and LCNEC of the prostate, exploring the influence of demographics, survival rates, treatment modalities, and various other factors on patient outcomes. Key conclusions include that the multivariate analysis showed the prognostic nomogram to indicate there was a slight increase in mortality for patients of Asian race and with tumors greater in size, a significant increase in death for patients of stage 3 tumors, and an increase in death for patients that underwent surgery and radiation. These findings offer a deeper understanding of these rare prostate cancer subtypes, with significant implications for future treatment plans and clinical practice.

## Figures and Tables

**Table 1:** Demographic factors.

Variable (*n* = 718)		Frequency (%)
Age (years)	<70	305 (42.5%)
	≥70	413 (57.5%)
Ethnicity	Unknown	2 (0.3%)
	White	534 (74.4%)
	Black	66 (9.2%)
	Asian or Pacific Islander	42 (5.8%)
	Hispanic	68 (9.5%)
	American Indian/Alaska Native	6 (0.8%)
Income (US$/year)	≤$75,000	388 (54.0%)
	≥$75,000	330 (46.0%)
Rural-Urban Continuum	Metropolitan	618 (86.1%)
	Rural	100 (13.9%)

**Table 2:** Tumor and Treatment Characteristics.

Variable (*n* = 718)		Frequency (%)
Prostate Histological Type	SCNEC	688 (95.8%)
	LCNEC	30 (4.2%)
Tumor Size	When known (*n* = 89) (B = 0.88)	
	<2.1 cm	15 (16.9%)
	2.1–4.0 cm	13 (14.6%)
	>4.1 cm	61 (68.5%)
Local Lymph Node Status	When Known (*n* = 58) (B = 0.79)	
	Positive	30 (51.7%)
	Negative	28 (48.3%)
Metastasis	Metastasis Status (*n* = 292) (B = 0.95)	
	Positive Metastases	211 (27.7%)
	Negative Metastases	81 (72.3%)
	# of Metastasis (*n* = 211) (B = 0.92)	
	Single Site	169 (57.9%)
	Multiple Sites	123 (42.1%)
	Site of Metastases (*n* = 211) (B = 0.92)	
	Bone	138 (47.3%)
	Brain	14 (4.8%)
	Liver	101 (34.6%)
	Lung	47 (16.1%)
	Distant Lymph Node	75 (25.7%)
	Other	29 (9.9%)
Underwent Treatment Modality	Chemotherapy	416 (57.9%) *
	Radiation	77 (10.7%)
	Surgery	207 (28.8%)

* All other cases chemotherapy status is unknown B = Sample power.

**Table 3:** Univariate and multivariate analysis for factors contributing to mortality.

Variables		Bivariate		Multivariate	
		Hazard Ratio (C.I. 95%)	*p*-Value	Hazard Ratio (C.I. 95%)	*p*-Value
Age		1.02 (1.01–1.03)	<0.001 *	1.02 (0.98–1.07)	0.365
Ethnicity ^#^					
Non-Hispanic White	869 (83.6%)	1		1	
Non-Hispanic Black	52 (5.0%)	0.86 (0.65–1.15)	0.306	1.10 (0.11–11.06)	0.936
Non-Hispanic Asian/PI	42 (4.0%)	1.07 (0.76–1.50)	0.706	0.69 (0.05–9.02)	0.776
Hispanic (All Races)	75 (7.2%)	0.67 (0.50–0.88)	0.005 +	0.52 (0.14–1.97)	0.335
Stage					
Localized	440 (42.3%)	1		1	
Regional	184 (17.7%)	1.70 (1.21–2.39)	0.002 *	0.60 (0.45–7.83)	0.693
Distant	154 (14.8%)	2.63 (2.00–3.53)	<0.001 *	0.39 (0.03–5.61)	0.487
Nodal Status					
Negative	164 (15.8%)	1		1	
Positive	53 (5.1%)	2.81 (1.49–5.30)	0.001 *	3.03 (0.79–11.52)	0.105
Chemotherapy Only					
No	714 (86.7%)	1		1	
Yes	326 (31.3%)	0.91 (0.77–1.07)	0.244	0.7 (0.00–1.43)	0.084
Surgery Only					
No	714 (86.7%)	1		1	
Yes	326 (31.3%)	0.93 (0.72–1.20)	0.575	0.02 (0.00–1.17)	0.059
Surgery + Chemotherapy					
No	394 (62.1%)	1		1	
Yes	394 (37.9%)	0.98 (0.75–1.29)	0.881	0.02 (0.00–1.19)	1.88
Radiation + Chemotherapy					
No	394 (62.1%)	1		1	
Yes	394 (37.9%)	0.79 (0.41–1.52)	0.473	0.03 (0.00–2.29)	0.113
Combination					
No	841 (80.9%)	1		1	
Yes	199 (19.1%)	0.75 (0.52–1.07)	0.107	0.02 (0.00–1.92)	0.093
Systemic Therapy					
No	332 (40.0%)	1		1	
Yes	497 (60.0%)	0.78 (0.63–0.96)	0.021 +	1.21 (0.10–15.17)	0.882

* Associated with worse survival outcomes; + Associated with better survival outcomes; ^#^ Non-Hispanic American Indian and Alaskan Native was removed due to having only 1 case, Significant for being associated with better outcomes.

## Data Availability

All data are publicly available and will be provided upon appropriate request from the corresponding author.

## Supplementary Materials

The following supporting information can be downloaded at: https://www.mdpi.com/article/10.3390/jcm13164874/s1, Figure S1: Overall Survival analysis by (a) income, (b) housing, (c) nodal status, and (d) metastasis. Table S1: Survival data of overall observed vs cause-specific; Table S2: Survival by treatment modality; Table S3: Survival by race; Table S4: Survival by stage; Table S5: Survival by histology.
