# Supplementary material for: Prognostic Nomogram Predicting Survival and Propensity Score Matching with Demographics and Comparative Analysis of Prostate Small Cell and Large Cell Neuroendocrine Carcinoma
Source: J Clin Med. 2024 Aug 18;13(16):4874. doi: 10.3390/jcm13164874 (PMC11355222; doi:10.3390/jcm13164874)

Supplemental Table S1. Survival data of overall observed vs cause-specific

Survival	Overall Observed% (C.I. 95%)	Cause specific survival% (C.I. 95%)
1 year	18.5% (17.0-20.0)	28.3% (26.3-30.3)
5 years	8.0% (6.8-9.2)	16.2% (14.3-18.1)

Supplemental Table S2. Survival by treatment modality

Survival	Chemotherapy Only* (C.I. 95%)	Surgery Only* (C.I. 95%)	Surgery + Chemotherapy%* (C.I. 95%)	Combination (C.I. 95%)
1 year	15.5% (13.2-17.8)	24.0% (19.9-29.9)	12.0% (7.7-16.3)	31.7% (23.7-39.7)
5 years	3.5% (2.1-4.9)	18.2% (13.6-22.8)	4.8% (1.7-7.9)	5.0% (1.0-9.0)

Supplemental table S3. Survival by race

Survival by race	White% (C.I. 95%)	Black% (C.I. 95%)	Hispanic (C.I. 95%)
1 year	17.2% (15.5-18.9)	22.6% (17.0-28.2)	28.1% (22.2-34.0)
5 years	7.3% (6.0-8.3)	11.9% (7.3-16.5)	12.2% (7.7-16.7)

*Both cohorts Asian or Pacific Islander & American Indian or Alaska Native were excluded due to limited power

Supplemental Table S4. Survival by stage

Survival by stage	Localized (C.I. 95%)	Regional (C.I. 95%)	Distant (C.I. 95%)
1 year	42.3% (36.4-48.2)	25.6% (21.0-30.2)	10.3% (8.6-12.0)
5 years	28.4% (22.9-33.9)	10.7% (7.4-14.0)	2.0% (1.1-2.9)

Supplemental Table S5. Survival by histology

Survival by stage	Small Cell (C.I. 95%)	Large Cell (C.I. 95%)
1 year	18.3% (16.7-19.9)	24.6% (16.0-33.2)

Supplemental Figure S1: Overall Survival analysis by (**a**) income, (**b**) housing, (**c**) nodal status, and (**d**) metastasis

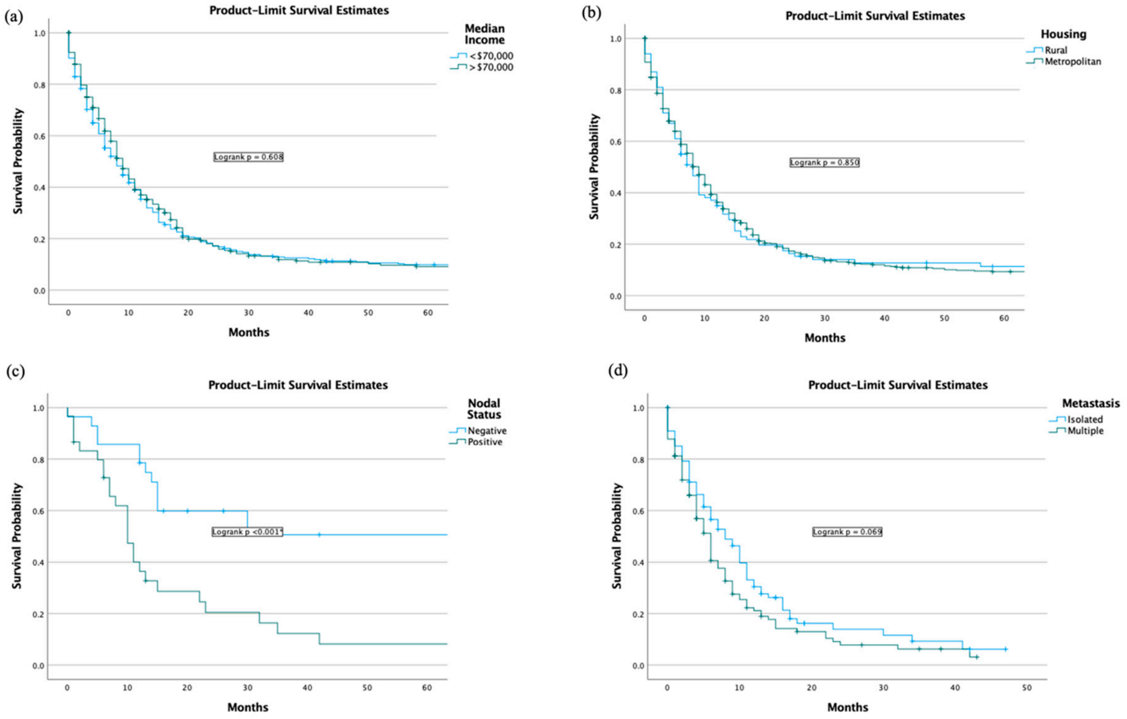

Supplement: Supplementary file 1 [file jcm-13-04874-s001.zip › jcm-3106286-Supplementary Materials.pdf]
